# A histopathological and biometric comparison between catfish (Pisces, Ariidae) from a harbor and a protected area, Brazil

**DOI:** 10.1186/s12999-014-0012-5

**Published:** 2014-12-14

**Authors:** Raimunda Nonata Fortes Carvalho Neta, Débora Batista Pinheiro Sousa, Zafira da Silva de Almeida, Débora Martins Silva Santos, Ligia Tchaicka

**Affiliations:** Department of Chemistry and Biology, State University of Maranhão (UEMA), São Luís, Maranhão Brazil; Postgraduate Program of Aquatic Resources and Fishery (PPGRAP/UEMA), State University of Maranhão, São Luís, Maranhão Brazil

**Keywords:** Biomonitoring, Biomarkers, *Sciades herzbergii*, *Bagre bagre*

## Abstract

**Background:**

Histopathological lesions and biometric variations in catfish species are statistically associated with chemical contaminant exposure. A histopathological and biometric database for the catfish *Sciades herzbergii* and *Bagre bagre* from São Luís Island (Port Area) and Caranguejos Island (Reference Area) is presented. Branchial and hepatic lesions were classified into three reaction patterns: 1) circulatory or inflammatory disturbances; 2) regressive changes; 3) progressive changes. This paper summarizes research efforts aimed at characterizing the biomonitoring potential of catfish from two islands in Brazil, which exhibit great habitat diversity and different levels of human intervention.

**Results:**

The weights and lengths of the catfish caught at the Port Area were smaller than those from the Reference Area. No histopathological lesions were observed in *S. herzbergii* examined at the reference site (Caranguejos Island). In contrast, 90% of *S. herzbergii* from sites located in the Port Area (São Luís Island) had one or more types of branchial and hepatic lesions. One or more of the five lesions were observed on 16 *B. bagre* from São Luís Island and Caranguejos Island.

**Conclusion:**

The utility of histopathological lesions and biometric data as sensitive indicators of the health of wild catfish populations has been demonstrated. *Sciades herzbergii* proved to be a better species for biomonitoring because it was more sensitive to the impacted site (Port Area) compared with the region relatively free of contaminants (Reference Area).

## Background

Histopathological analysis in fish has already been tested and proposed as a sensitive tool for the monitoring of environmental contamination in natural water bodies [1]. Fish diseases, biometric data, and histopathology are increasingly being used as indicators of environmental stress because they provide a definite biological end-point of historical exposure [2].

Biomarker analysis has been shown to be adequate for ascertaining the toxic effects of a given toxicant [3]. Biomarkers have been defined by measurable modifications at the molecular, biochemical, cellular, physiological, and behavioral levels, revealing the exposure of a given organism to xenobiotics [4].

Gill lesions are used as sensitive biomarkers of environmental impacts on fish, and many researchers have recognized that histopathological examination is a valuable tool for assessment of environmental impacts on fish populations [5] because morphologic alterations can occur as gills are in permanent contact with the environment [6]. The detection of early warning signals through branchial and hepatic lesions in fish is ecologically relevant, economically beneficial, more efficient, and has the potential to be used as a type of biomarker [7].

In São Luís Island (São Marcos Bay, Maranhão), the catfish *Sciades herzbergii*, a benthic fish common to Brazil coastal areas, has been identified as a model species for investigating the effects of contamination in estuarine systems [3]. *Bagre bagre*, another catfish, resides in both contaminated and clean estuarine environments around the São Marcos Bay. This species is particularly suitable for this research because of its commercial importance and sedentary lifestyle.

São Marcos Bay is an important fishing location and has the most important harbor in northeastern Brazil. However, in the last decade, chemical contamination from industrial waste has posed an increasing threat to the health of aquatic organisms [7,8]. In contrast, Caranguejos Island constitutes a wildlife reserve in São Marcos Bay that covers a large mangrove area [8]. The increasing emphasis in Brazil on monitoring estuarine and marine ecosystems has highlighted the need to deploy appropriate biological measurements for these locations [8]. This study gathers and compares biometric data and histopathological lesions of *S. herzbergii* and *B. bagre* from ecologically diverse ecosystems on two distinct Islands in São Marcos Bay, Brazil.

## Results and discussion

### Environmental parameters

The water from the two sites (São Luís and Caranguejos Islands) exhibited distinct characteristics and contamination profiles (Table 1). The concentrations of Al, Cd, Pb, Cr, Fe, and Hg, in São Luís Island were higher than the acceptable limit by national Brazilian standards (CONAMA, Conselho Nacional do Meio Ambiente). The average water surface temperatures were constant during both periods analyzed. Salinity was uniform in both areas sampled, being lower during the rainy season in both areas. Dissolved oxygen and the saturation of dissolved oxygen were always lower in the contaminated area. The values for pH were constants for both areas. The habitats of each of the different sites were identical in aquatic plants, substrate, and fauna.

**Table 1:** **Physical and chemical analysis of water at two sites in São Marcos Bay (Brazil)**

**Parameters**	**Reference (A1)**		**Contaminated (A2)**	
	**Dry period** *	**Rainy period** ^**y**^	**Dry period** *	**Rainy period** ^**y**^
Temperature (°C)	30.5	29.5	30.5	29.5
Salinity (UPS)	15	14	19	18
pH	7.98	7.99	7.94	7.94
OD (mg/L O_2_)	6.0	6.1	4.9	5.1
Aluminum (mg/L)	0.02	0.02	0.1	0.1
Cadmium (mg/L)	0.001	0.001	0.007	0.006
Lead (mg/L)	0.001	0.000	0.02	0.02
Chromium (mg/L)	0.08	0.07	0.02	0.1
Iron (mg/L)	0.01	0.01	0.07	0.07
Mercury (mg/L)	0.000	0.000	0.001	0.001

*Dry period = August 2011; ^y^Rainy period = February 2012.

Abiotic factors such as water temperature, conductivity, pH, and dissolved oxygen can change the fish richness and assemblage composition [8-10], which can also be affected by anthropogenic impacts [11]. The similar pH values recorded for the two analyzed areas indicate a dynamic region where the winds, tides, and river discharges determine a high load of particulate matter. All heavy metals concentrations (Al, Cd, Pb, Cr, Fe, Hg) in water collected from the potentially contaminated area were significantly higher than water from the reference area. Previous studies on sediment and water in the harbor area showed significantly higher levels of mercury and chrome, which confirms that the port area in São Marcos Bay is a site with high exposure risks for some contaminants [3,7,8]. Öztürk et al. [12] have determined the level of heavy metals in various tissues of the Cyprinus carpio species and they reported that cadmium, chromium, nickel, and lead concentrations exceeded the tolerable values provided by international institutions. These authors have reported that heavy metal concentrations in gill samples can decrease in the sequence: Fe (0.15 mg/kg) > Cu (3.94 mg/kg) > Pb (3.11 mg/kg) > Cr (1.61 mg/kg) > Cd (0.15 mg/kg), and for the liver as Fe (94.27 mg/kg) > Cu (9.73 mg/kg) > Pb (3.42 mg/kg) > Cr (0.83 mg/kg) > Cd (0.79 mg/kg).

### Biometric data

Biometric parameters (total length - Lt, furcal length - Lf, total weight – Wt and gonad weight - Wg) of collected fish are showed in Tables 2 and 3. As expected, most populations of catfish considered in this study are highly heterogeneous, with lengths and weights deviating from the reference sample. At the reference site (A1), catfish were bigger (P < 0.05) than those from the harbor area (A2). Fish across all gonadal stages were found in the reference area, but juveniles (GS1) were not found in the potentially contaminated site. The gonadosomatic index (GSI) was also higher in the reference site than in the contaminated site during both periods (P < 0.05). In a similar study, the GSI in *S. herzbergii* from the potentially contaminated site (São Marcos Bay) was significantly lower than in control fish during all the phases of the gonadal cycle [3-5]. Carvalho-Neta et al. [3] have shown a decrease in the GSI of catfishes from harbor areas in Brazil, and have suggested this decrease can result in abnormal gonadal development in the form of delayed maturation, high levels of atresia, or intersexuality in fish.

**Table 2:** **Biometric data and gonadosomatic index of** ***S. herzbergii*** **caught in São Marcos Bay, Maranhão, Brazil**

**Sites**	**Gender**	**L**_**t**_ **(cm)**	**L**_**f**_ **(cm)**	**W**_**t**_ **(g)**	**W**_**g**_ **(g)**	**GSI**
Reference (A1)	Female	29.85 ± 2.72*	26.29 ± 2.98*	283.45 ± 57.34*	7.936 ± 0.361	2.68 ± 0.26*
	Male	28.85 ± 2.81*	27.29 ± 2.21*	281.25 ± 37.54*	5.434 ± 0.251	1.58 ± 0.12*
Harbor (A2)	Female	20.88 ± 3.22	19.45 ± 2.74	108.50 ± 77.90	0.629 ± 0.447	0.08 ± 0.063
	Male	18.28 ± 8.72	16.25 ± 2.92	105.31 ± 55.60	0.129 ± 0.047	0.02 ± 0.013

*Indicates significant difference between sites (P < 0.05). Total number of catfish = 60. Number of female catfish in: A1 = 15; A2 = 15. Number of male catfish in: A1 = 15; A2 = 15. Biometric data: L_t_ (total length); L_f_ (fork length); Wg (gonad weight), and GSI (gonadosomatic index). cm (centimeter); g (gram).

**Table 3:** **Biometric data and gonadosomatic index of** ***B. bagre*** **caught in São Marcos Bay, Maranhão, Brazil**

**Sites**	**Gender**	**L**_**t**_ **(cm)**	**L**_**f**_ **(cm)**	**W**_**t**_ **(g)**	**W**_**g**_ **(g)**	**GSI**
Reference (A1)	Female	26.32 ± 1.82*	24.29 ± 2.01*	222.65 ± 14.34*	5.895 ± 0.351	1.51 ± 0.85*
	Male	27.21 ± 1.31*	25.29 ± 1.21*	228.15 ± 13.54*	1.434 ± 0.451	0.58 ± 0.11*
Harbor (A2)	Female	20.53 ± 4.44	18.45 ± 2.74	104.50 ± 57.90	0.729 ± 0.57	0.08 ± 0.02
	Male	18.28 ± 8.72	16.05 ± 3.92	102.11 ± 35.60	0.129 ± 0.08	0.03 ± 0.01

*Indicates significant difference between sites (P < 0.05). Total number of catfish = 60. Number of female catfish in: A1 = 15; A2 = 15. Number of male catfish in: A1 = 15; A2 = 15. Biometric data: L_t_ (total length); L_f_ (fork lengt); Wg (gonad weight), and GSI (gonadosomatic index). cm (centimeter); g (grama).

### Branchial and hepatic lesions

No deformation of the gills and livers were observed in S. *herzbergii* collected in the reference area. However, more than half (90%) of catfish (S. *herzbergii*) collected in the contaminated area showed moderate to severe gill and liver damage (Table 4). No ectoparasites or endoparasites were observed in *S. herzbergii*, which suggests that other environmental parameters may have been the cause of observed gill lesions. As opposed to what was observed in *S. herzbergii*, more than 86.33% of *B. bagre* individuals showed histopathological alterations in both areas (Table 4). Several ectoparasites were observed in *B. bagre*. The migratory behavior and parasitosis observed in *B. bagre* seems to explain the number of lesions observed in individuals from both areas [13-19]. However, *S. herzbergii* is a resident and non-migratory estuarine species of catfish [16,17].

**Table 4:** **Observed pathologies and their importance factor (w) and percentages (%) in catfish from São Marcos Bay**

**Target organ**	**Reaction pattern**	Alteration*	**w**	**%** ***S. herzbergii***	**%** ***B. bagre***
Liver	Inflammatory response	*Profusion and dilation of blood vessels (L1)*	1	A1 = 0/A2 = 93.33	A1 = 43.33/A2 = 66.66
		*Presence of melanomacrophages (L2)*	1	A1 = 0/A2 = 93.33	A1 = 32.33/A2 = 66.66
	Regressive changes	*Nuclear pleomorphisms (L3)*	2	A1 = 0/A2 = 66.66	A1 = 23.33/A2 = 66.66
		*Necrosis (L4)*	3	A1 = 0/A2 = 90.00	A1 = 16.66/A2 = 86.66
	Progressive changes	*Lipidosis (L5)*	1	A1 = 0/A2 = 66.66	A1 = 16.66/A2 = 86.66
		*Eosinophilic hepatocellular alteration (L6)*	2	A1 = 0/A2 = 86.66	A1 = 16.66/A2 = 66.66
		*Granulomatous lesions (L7)*	2	A1 = 0/A2 = 86.66	A1 = 43.33/A2 = 90.00
		*Presence of eosinophilic bodies (L8)*	2	A1 = 0/A2 = 90.00	A1 = 66.66/A2 = 86.66
Gills	Circulatory disturbances	*Lamellar capillary aneurism (L9)*	1	A1 = 0/A2 = 63.33	A1 = 16.66/A2 = 96.66
	Regressive changes	*Epithelial lifting (L10)*	1	A1 = 0/A2 = 63.33	A1 = 90.00/A2 = 86.66
		*Epithelial desquamation (L11)*	1	A1 = 0/A2 = 23.33	A1 = 23.33/A2 = 43.33
		*Deformation of lamellae (L12)*	1	A1 = 0/A2 = 90.00	A1 = 16.66/A2 = 66.66
		*Mucous (goblet) cell degeneration (L13)*	2	A1 = 0/A2 = 90.00	A1 = 33.33/A2 = 86.00
	Progressive changes	*Hypertrophy of squamous epithelia (L14)*	1	A1 = 0/A2 = 23.33	A1 = 23.33/A2 = 16.66
		*Lamellar fusion (L15)*	1	A1 = 0/A2 = 93.33	A1 = 43.33/A2 = 93.33
		*Epithelial hyperplasia (L16)*	2	A1 = 0/A2 = 93.33	A1 = 16.66/A2 = 86.33

A1 = reference; A2 = contaminated area.

*Only persistent pathologies within an organ were scored as observed. Alterations that did not qualify as representative of the overall organ condition were disregarded, being considered as natural variations.

The histopathological examination performed in the gill epithelium of catfish *S. herzbergii* could clearly differentiate the region of Caranguejos Island (reference – A1) and the harbor site (contaminated – A2). *Sciades herzbergii* is a benthic and resident species of catfish. The great number of moderate and severe branchial and hepatic lesions indicates that harbor fish are stressed by the pollutants. Branchial and hepatic lesions such as the profusion and dilation of blood vessels, presence of melanomacrophages, and epithelial lifting are examples of defense mechanisms [13,14].

In São Marcos Bay, the integrated analysis of morphological lesions and biometric data were useful because histopathological alterations were detected only in catfish of the Port area [7]. Furthermore, in that area, GSI was very low and juvenile fish were not found. Carvalho-Neta et al. [8] observed the same situation at São Marcos Bay and they suggested a method of correlating branchial and hepatic lesions with biometric data for biomonitoring in this region.

**Figure 1:**
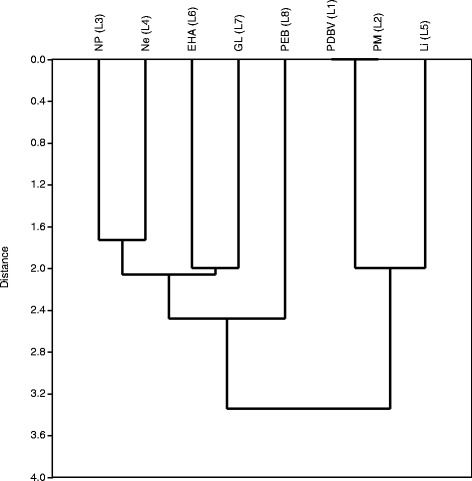
**Joining tree of observed lesions in the liver of** ***Sciades herzbergii*** **from a port area in São Marcos Bay, Brazil.** Distances were obtained from presence and absence data and estimated as 1-Pearson r. Joining is based on unweighted pair-group averages.

**Figure 2:**
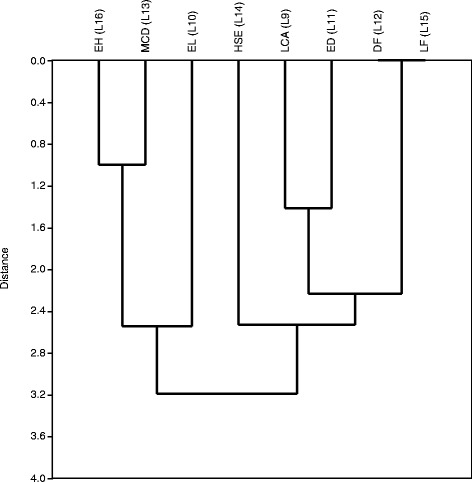
**Joining tree of observed lesions in the gills of** ***Sciades herzbergii*** **from a port area in São Marcos Bay, Brazil.** Distances were obtained from presence and absence data and estimated as 1-Pearson r. Joining is based on unweighted pair-group averages.

As the data on *S. herzbergii* lesions were more consistent for differentiating the two regions of São Marcos Bay, we made correlations between these morphological changes. Cluster analyses derived correlations between lesions (Figures 1 and 2). Regarding hepatic lesions, three groups of lesions were conspicuous. The first group comprises profusion and dilation of blood vessels (L1), presence of melanomacrophages (L2) and lipidosis (L5); the second group comprises nuclear pleomorphisms (L3) and necrosis (L4); and the third group comprises eosinophilic hepatocellular alteration (L6) and granulomatous lesions (L7). These clusters indicate prolonged physiological disturbances that led to glycogen depletion and lipid storage. These lesions, like melanomacrophages associated with lipidosis (intracellular lipid storage in large vacuoles) have been observed in wild fish from sites contaminated by mixtures of xenobiotics [20,21].

In gills, the strongest correlation was observed between deformation of lamellae (L12) and lamellar fusion (L15). The second group comprises epithelial hyperplasia (L16) and mucous cell degeneration (L13). The third group was formed by lamellar capillary aneurism (L9) and epithelial desquamation (L11). A similar joining tree was observed in the gills of juvenile *Solea senegalensis* exposed to contaminated estuarine sediments, indicating exposure to heavy metals [22]. This result suggests that gills are more susceptible to the acute effects of exposure to contaminants and livers are subjected to the chronic effects of xenobiotics.

## Conclusion

The present study demonstrates that *S. herzbergii* has great suitability for monitoring estuarine areas, especially on the basis of branchial lesions (acute effects), because the species shows greater sensitivity to an impacted area (harbor) compared with one relatively free of contaminants (reference).

## Methods

### Study areas

**Figure 3:**
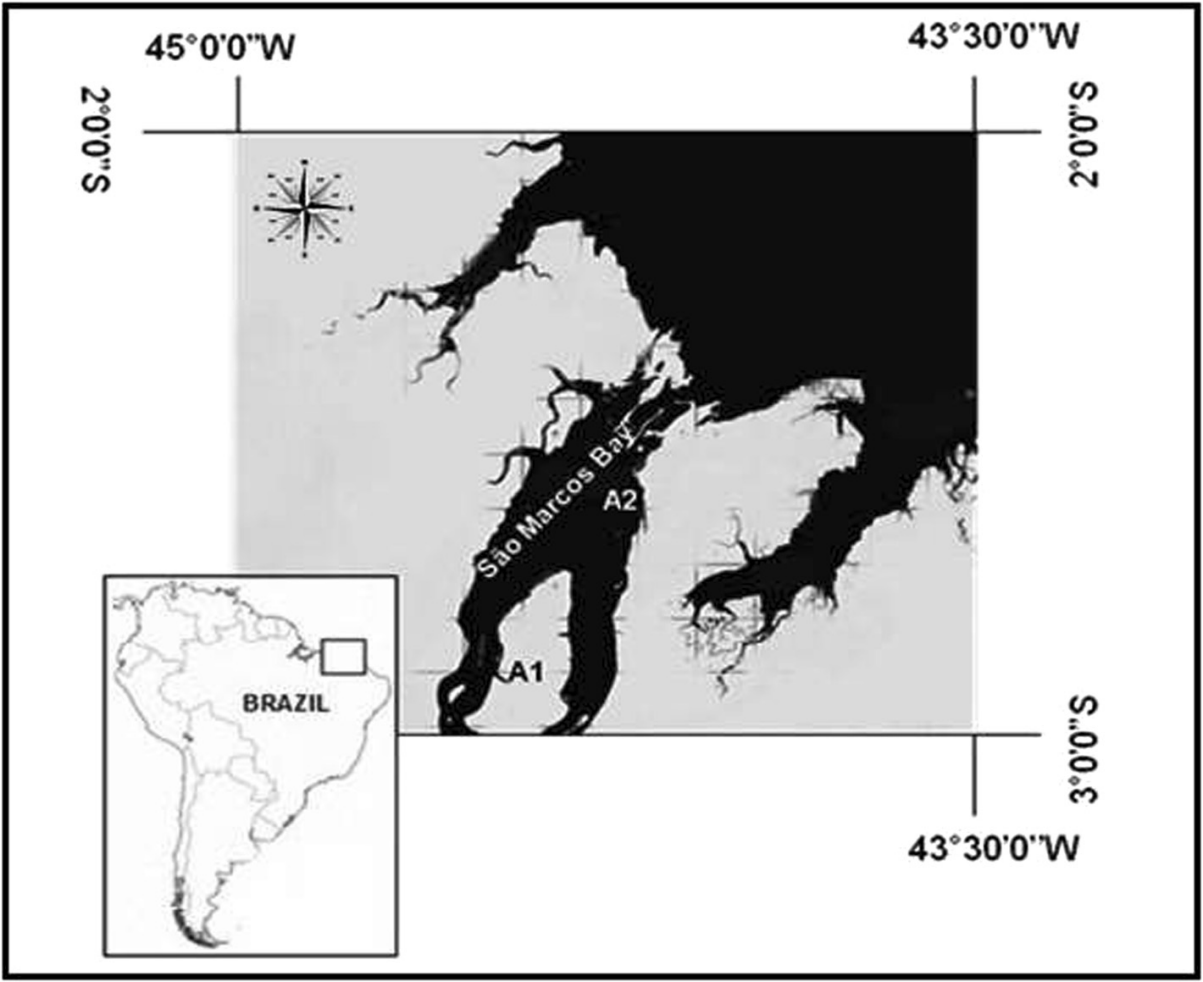
**São Marcos Bay, Maranhão Brazil.** A1 = Crab Island. A2 = São Luís Island.

We studied two sites along São Marcos Bay (Figure 3), Maranhão, Brazil. The first site (A1) is located in Crab Island (02°49’ 06”S and 044°29’05”W) and was used as a reference site because it is a natural reserve. The second site (A2) is located in the Port of Itaqui (02°43’14”S and 044°23’35”W), and was used to represent an impacted area because it receives agricultural, industrial, and domestic sewage effluents as well as harbor-related residuals [3].

The Port of Itaqui is located to the west coast of São Luis Island. The infrastructure of this port comprises a 1,616 m extension of quaysides. Fuel, iron, ore, manganese, grains, fertilizers, petroleum, bauxite, and alumina are shipped in this port [23].

The Caranguejos Island, situated at a distance of 30 km from the Itaqui port in São Marcos Bay, is an environment-protected area created by the government of the Maranhão. This region is uninhabited and occupies an area of 345.08 km^2^, containing the largest continuous stretch of mangrove in Maranhão [16].

### Sample collection

Specimens of *S. herzbergii* (common name in English is pemecou sea catfish and in Portuguese is bagre guribu) and *B. bagre* (common name in English is coco sea catfish and in Portuguese is bagre-bandeirado) were collected from three streams, about a kilometer from each other at each site (A1 and A2). Fish were captured during the rainy period (August 2011) and the dry period (February 2012). The capture of fish was authorized by the local environmental agency (Secretaria de Estado de Meio Ambiente e Recursos Naturais – SEMA), number 001/12. Thirty fish in the potentially impacted area and 30 fish in the reference area were randomly sampled. The sexes for these fish by season were: A1) eight male and seven female fish in the dry season; seven male and eight female fish in the wet season; A2) nine male and six female fish in the dry season; eight male and seven female fish in the wet season. Fish were killed by trans-spinal dissection and immediately transported in an isothermal box (equipped with refrigeration system) to the laboratory.

Water samples were collected from the port area and the reference area for determination of contaminant levels. The concentrations of metals in water were determined using flame atomic absorption spectrophotometry (AAS), with a nitrous oxide-acetylene flame [24].

### Biometric data and somatic index

The total length (Lt), standard length (Ls), furcal length (Lf), total weight (Wt), and gonad weight (Wg) of each fish were recorded. The macroscopic classification of the gonadal stage (GS) was also undertaken: GS1 (immature), GS2 (maturing or at rest), GS3 (mature), and GS4 (spent), following the scale given by Carvalho-Neta and Castro [16]. The gonadosomatic index (GSI) was calculated in accordance with Vazzoler [25].

### Histopathological analysis

Fish were dissected and their gills and livers were fixed immediately in 10% formalin. The gills and livers of each individual were dehydrated in a progressive series of ethanol dilutions and embedded in paraffin. Sections were stained with hematoxylin and counterstained with alcoholic eosin for structural analysis of gills and liver. Four tissue sections from each fish were examined by Zeiss light photomicroscope. Histopathological lesions were classified according to Bernet et al. [1] into three reaction patterns: 1) circulatory disturbances; 2) regressive changes; 3) progressive changes. We considered 16 types of histopathological lesions. Every alteration has an importance factor (w) from 1 to 3 where 1 = minimal pathological importance (the lesion is easily reversible); 2 = moderate pathological importance (the lesion is reversible in most cases); 3 = marked pathological importance (the lesion is generally irreversible) [1].

### Statistical analysis

Results were expressed as mean ± standard deviation. T-tests and one-way analysis of variance were used to test for significant differences between groups (on the contaminated and the reference site) and only p < 0.05 was accepted as significant. Biometric data (LT, LS, LF, WT, WG, and GSI) were calculated using the mean values observed for each parameter. Cluster analysis was based on correlation matrices by computing Pearson’s r statistic. Pairwise correlations were obtained through the Spearman’s rank-order correlation “p”. The significance level was set at p = 0.05.

## Abbreviations

GSI: Gonadossomatic Index
Lt: Total length
Lf: Fork length
Wg: Gonad weight
cm: Centimeter
g: Gram
